# Perception of Current and Future Commitment of Medical Teachers to the Varied Roles of a Teacher: An Observational Study

**DOI:** 10.7759/cureus.45824

**Published:** 2023-09-23

**Authors:** Shakti K Yadav, Shikha Para, Alekh Verma, Ruchika Gupta, Namrata Sarin, Sompal Singh

**Affiliations:** 1 Pathology and Laboratory Medicine, All India Institute of Medical Sciences, Bhopal, Bhopal, IND; 2 Obstetrics and Gynecology, Gandhi Medical College, Bhopal, IND; 3 Pathology, North Delhi Municipal Corporation Medical College and Hindu Rao Hospital, New Delhi, IND; 4 Cytopathology, National Institute of Cancer Prevention and Research, Noida, IND

**Keywords:** information provider, teacher, role model, resource developer, planner

## Abstract

Background

Medical education is gradually moving towards self-directed learning, thus the roles of a teacher have assumed wider dimensions than before. The awareness of these roles among medical teachers has been studied in several countries, but no study on the awareness of these roles among Indian medical faculty has been found. The aim of this research was to assess the current and future commitment perception of the roles of a teacher among Indian medical faculty.

Methods

A cross-sectional questionnaire-based survey regarding the 12 roles of a teacher, as defined by Harden and Crosby, was conducted among medical teachers in a tertiary-level hospital and medical college. The questionnaire consisted of three categories: importance in medical teaching, current commitment, and preferred future commitment to these roles, all measured on a five-point Likert scale.

Results

The highest mean scores were given to the roles of learning facilitator and on-the-job role model. In contrast, the lowest scores were designated to the production of study guides. Interestingly, the teachers’ current commitment to roles such as curriculum planner and course organizer was found to be low. A significant difference was observed between the three categories for the majority of the roles. Younger faculties showed significant difference among categories, while the senior professors did not show significant variations across the roles.

Conclusion

This study of Indian medical teachers emphasizes the decreased importance attributed to roles like curriculum planning and course organization. Further studies in other developing countries are essential to understand this issue more comprehensively.

## Introduction

Medical education has undergone a dramatic change in the last few years with the inclusion of newer methods of learning as well as assessment [1]. This has in turn led to redefinition of the roles of a medical teacher to conform to the concept of student-based learning. Medical teaching is more than just classroom or bedside teaching - it includes role modeling, mentoring, curriculum planning and other roles, defined best by Harden and Crosby [1,2]. However, awareness among medical teachers of their various roles seems lacking in one aspect or the other, mainly due to the absence of formal teachers’ training programs for incumbents [3]. This awareness is also modulated by the teachers’ own educational background, their teacher’s way of teaching and the educational environment of the institution they are employed with [4].

The 12 roles of a teacher outlined by Harden and Crosby with eloquent description of each role have stood the test of time since their first publication in 2000. This is evident by the number of studies conducted in different specialities and institutions. A study among physiotherapy students and teachers showed that roles like demonstrator, assessor, knowledge provider, mentor and facilitator were thought to be important for learning [5]. Another study from Pakistan showed that the participating medical teachers perceived the roles in a traditional fashion as lecturer/clinical teacher, role model, learning facilitator and assessor [6]. An extensive review of the available literature revealed only one Indian study and that too among dental teachers about their perception of the roles of a teacher [7]. However, we could not find any study conducted among teachers in a medical college in India. Hence, this study was designed with the aim to analyze the medical teachers’ perception of the 12 roles of a teacher and assess their current as well as future personal commitment to each of these roles, and also to investigate how medical teachers across different ranks (senior residents, assistant professors, associate professors, and professors) perceive the roles and how their current and future personal commitments to these roles differ.

## Materials and methods

This was a cross-sectional descriptive and analytical study conducted over a period of three months (April to June 2018) at a tertiary care hospital and associated medical college in Delhi. The ethical approval was granted by the institutional ethics committee.

The roles of teachers, as defined by Harden and Crosby [1] in AMEE guide no. 20, were utilized and a questionnaire, similar to that used by Harden and Crosby in their study, was designed incorporating these roles. The questionnaire had 12 questions pertaining to the individual 12 roles of teachers. It was implemented in three categories as described by Harden and Crosby in their seminal paper: Importance of the roles to the medical school teaching program (Category 0), teachers’ current personal commitment to the individual roles (Category 1) and their preferred future commitment (Category 2). For each of these questions, the participants were asked to provide their answers on a five-point Likert scale: (1) None, (2) Little, (3) Some, (4) Considerable, and (5) Great. The questionnaire was pre-tested on five teachers in the college for clarity of questions and understanding of the terms used. These five teachers were not included in the subsequent study and analysis.

The questionnaire was administered to 98 teachers in our college, after taking their informed consent to be a part of the study. The teachers included senior residents, assistant professors, associate professors, professors as well as medical specialists involved in undergraduate medical teaching. The data was anonymized and analyzed using SPSS version 23.0 (IBM Corp., Armonk, NY, USA) to determine the mean score for each role and comparison of the three categories. Shapiro-Wilk test was used to check for the normality of data. Non-parametric tests (Mann-Whitney U test) were used to analyze the data that was non-normal in distribution and intercategory comparison.

## Results

The response rate was 100% with all 98 teachers providing completed questionnaire, out of which senior residents were 65, assistant professors were 23, and senior professors (including associate professor and professor) were 10. The mean age of the participants was 40.46 years (±6.8 years) with a male:female ratio of 1.27:1. The teachers had a teaching experience ranging from three to eight years. The questionnaire utilized in this study is included in Table 1 along with the results.

**Table 1:** Questionnaire and Mean scores of 12 roles of a teacher in three categories in the present study IQR: Interquartile range

Teachers’ Role		Importance to medical school teaching programme (Category 0)		Current personal commitment (Category 1)		Preferred personal future commitment (Category 2)	
		Median	IQR	Median	IQR	Median	IQR
Information provider	(1) Lecturer in classroom setting	4	1	4	0.25	5	1
	(2) Teacher in clinical or practical class setting	5	1	4	1.25	4	1
Role model	(3) On-the-job role model (e.g. in clinics, ward rounds, etc.)	5	0.25	3.5	1	5	1
	(4) Role model in the teaching setting	5	1	3.5	1	5	1
Facilitator	(5) Mentor, personal adviser or tutor to a student or group of students	5	1	4	1	4.5	1
	(6) Learning facilitator, e.g., supporting students’ learning in problem-based -learning small groups in the laboratory, in the integrated practical class sessions or in the clinical setting	5	1	3	1	4	1
Examiner	(7) Planning or participating in formal examinations of students	4	1	4	1	4	1
	(8) Curriculum evaluator evaluation of the teaching programme and the teachers	4	1	3.5	3	4	2
Planner	(9) Curriculum planner, participating in overall planning of the curriculum, through, for example, curriculum planning committees such as the Undergraduate Medical Education Committee	4.5	1	1	2.25	4	1.25
	(10) Course organizer, responsibility for planning and implementing a special course within the curriculum. This may, for example, relate to one system or one theme, or to a special study module	4	1	2	2.25	4	1.25
Resource developer	(11) Production of study guides to support students’ learning in the course	4	2	4	2.5	4	2
	(12) Developing learning resource materials in the form of computer programmes, videotape or print which can be used as adjuncts to the lectures and other sessions	5	1	3	1.25	4	1

Perception of the importance of roles in medical teaching

The table of median scores (Table 1) shows that all the roles were considered important with some differences between them. The median scores for the lecturer in the classroom, the role of an examiner or planner as well as production of study guides were somewhat lower than the other roles in the perceived importance to the medical school teaching programme.

Current & future personal commitments to defined roles

The participant teachers’ current and future personal commitment to each individual’s role was seen to differ in some respects (Table 1). Among the current commitments, the roles of lecturer in the classroom, teacher in clinical/practical class, mentor or tutor, planning formal examinations and preparing study guides received the highest median scores while curriculum planning had a median score of 1. On the other hand, the teachers’ future personal commitment to the various roles did not differ much from each other.

Statistical analysis

Test for normality of data revealed that neither of the categories (0, 1, 2) had normal distribution of the data. Non-parametric Kruskal-Wallis test between the categories (0, 1 and 2) showed a significant difference in the teachers’ understanding of the importance of these roles in medical school teaching and their current or preferred future commitments (Table 2), except for the role as lecturer in the classroom setting (p = 0.087).

**Table 2:** Result of non-parametric Kruskal-Wallis test for comparison between test categories *P-value <0.05 – significant

	Chi-square	df	P-value*
Q1	4.881	2	0.087
Q2	11.570	2	0.003
Q3	23.293	2	<0.001
Q4	31.196	2	<0.001
Q5	27.222	2	<0.001
Q6	45.620	2	<0.001
Q7	11.220	2	0.004
Q8	18.011	2	<0.001
Q9	34.252	2	<0.001
Q10	34.944	2	<0.001
Q11	5.707	2	0.058
Q12	18.682	2	<0.001

Further, category-wise differences were elicited using the Mann-Whitney test between two categories due to the non-normal distribution of the data (Table 3). This table shows that though the highest importance was given to the role of lecturer in the classroom in all the categories, the participant teachers’ preferred future commitment was higher than the current commitment (1 vs 2, P=0.039). For the remaining 11 roles, a significant difference was noted between categories 0 and 1 and between 1 and 2. Between categories 0 and 2, a significant difference was noted for roles as learning facilitator, curriculum planner and course organizer, with preferred future commitment to these roles being lower than the perceived importance of these roles in medical school teaching.

**Table 3:** Results of inter-category comparison (P-values) of mean scores for teachers’ roles 0: Importance to medical school teaching programme, 1: Current personal commitment, 2: Preferred personal future commitment, *Mann-Whitney U test

		0 vs 1	1 vs 2	0 vs 2
Q1	Lecturer in classroom setting	0.101	0.039	0.53
Q2	Teacher in clinical or practical class setting	0.001	0.045	0.182
Q3	On-the-job role model	<0.001	<0.001	0.324
Q4	Role model in the teaching setting	<0.001	<0.001	0.668
Q5	Mentor, personal adviser or tutor to a student or group of students	<0.001	<0.001	0.321
Q6	Learning facilitator	<0.001	<0.001	0.046
Q7	Planning or participating in formal examinations of students	0.012	0.002	0.447
Q8	Curriculum evaluator evaluation of the teaching programme and the teachers	<0.001	0.003	0.248
Q9	Curriculum planner, participating in overall planning of the curriculum	<0.001	<0.001	0.013
Q10	Course organizer, responsibility for planning and implementing a special course within the curriculum	<0.001	<0.001	0.019
Q11	Production of study guides to support the students’ learning in the course	0.03	0.055	0.864
Q12	Developing learning resource materials in the form of computer programmes, videotape or print which can be used as adjuncts to the lectures and other sessions	<0.001	0.001	0.194

Analysis was also performed for difference in the scores of various categories as per age and gender of the participant teachers. Spearman rank correlation test did not show any significant correlation between the scores of various roles of a teacher and the age of the participant. Similarly, the scores of various roles for categories 0, 1 and 2 did not differ according to gender (Mann-Whitney U test, all P values >0.05).

Intercategory comparison among teacher subcategories

On comparing the scores for each category according to teacher type, some key findings came out. For the role of Information Provider, we found a statistically significant variation among Senior Residents. Specifically, the importance ascribed to category 0 compared to their current and future commitment had a p-value of 0.03 and 0.02, indicating a significant difference. This further emphasizes the evolving nature of commitment to this role in the new teachers. Similarly, for the role model category, senior residents showed significant differences between the perceived importance and both current and future commitments, with p-values of 0.01 and 0.03, respectively. Furthermore, a statistically significant disparity was observed between their current and future commitments, with a p-value of 0.02, suggesting a tendency to increase role commitment over time. Similarly, senior residents showed an increased inclination towards the role as facilitator more actively in the future. For the role as resource developer senior residents showed significant variations between categories 0 and 1, and 1 and 2, suggesting that they foresee an evolution in their commitment to this role as well (Table 4).

**Table 4:** Intercategory comparison for teachers' subcategories. Mann-Whitney U Test

Roles	Teacher Types	Category Comparison	Median (IQR)	P-value*
Information Provider	Senior Residents	0 vs 1	4.5 (1) vs 4 (0.5)	0.03
		0 vs 2	4.5 (1) vs 5 (1)	0.02
		1 vs 2	4 (0.5) vs 5 (1)	0.04
	Assistant Professors	0 vs 1	5 (0.5) vs 4 (0.5)	0.07
		0 vs 2	5 (0.5) vs 5 (1)	0.09
		1 vs 2	4 (0.5) vs 5 (1)	0.05
	Senior Professors	0 vs 1	5 (0.5) vs 5 (0.25)	0.11
		0 vs 2	5 (0.5) vs 5 (0.5)	0.14
		1 vs 2	5 (0.25) vs 5 (0.5)	0.10
Role Model	Senior Residents	0 vs 1	4.5 (1) vs 3.5 (1.25)	0.01
		0 vs 2	4.5 (1) vs 5 (1)	0.03
		1 vs 2	3.5 (1.25) vs 5 (1)	0.02
	Assistant Professors	0 vs 1	5 (0.5) vs 4 (1)	0.06
		0 vs 2	5 (0.5) vs 5 (0.5)	0.08
		1 vs 2	4 (1) vs 5 (0.5)	0.04
	Senior Professors	0 vs 1	5 (0.5) vs 4.5 (1)	0.09
		0 vs 2	5 (0.5) vs 5 (0.5)	0.12
		1 vs 2	4.5 (1) vs 5 (0.5)	0.07
Facilitator	Senior Residents	0 vs 1	5 (1) vs 4 (1.25)	0.02
		0 vs 2	5 (1) vs 4.5 (1)	0.03
		1 vs 2	4 (1.25) vs 4.5 (1)	0.01
	Assistant Professors	0 vs 1	4.5 (1) vs 4 (1)	0.07
		0 vs 2	4.5 (1) vs 4.5 (1)	0.09
		1 vs 2	4 (1) vs 4.5 (1)	0.05
	Senior Professors	0 vs 1	5 (1) vs 4.5 (1)	0.08
		0 vs 2	5 (1) vs 5 (1)	0.13
		1 vs 2	4.5 (1) vs 5 (1)	0.06
Examiner	Senior Residents	0 vs 1	4 (1) vs 4 (1)	0.12
		0 vs 2	4 (1) vs 4 (1)	0.14
		1 vs 2	4 (1) vs 4 (1)	0.11
	Assistant Professors	0 vs 1	4 (0.75) vs 3.5 (1)	0.09
		0 vs 2	4 (0.75) vs 4 (1)	0.07
		1 vs 2	3.5 (1) vs 4 (1)	0.08
	Senior Professors	0 vs 1	4.5 (0.5) vs 4 (0.75)	0.13
		0 vs 2	4.5 (0.5) vs 4 (1)	0.15
		1 vs 2	4 (0.75) vs 4 (1)	0.11
Planner	Senior Residents	0 vs 1	4 (1.5) vs 2 (1.75)	0.01
		0 vs 2	4 (1.5) vs 4 (1.25)	0.02
		1 vs 2	2 (1.75) vs 4 (1.25)	0.01
	Assistant Professors	0 vs 1	4.5 (1) vs 2.5 (1)	0.05
		0 vs 2	4.5 (1) vs 4 (1)	0.07
		1 vs 2	2.5 (1) vs 4 (1)	0.03
	Senior Professors	0 vs 1	4.5 (1) vs 3 (1)	0.08
		0 vs 2	4.5 (1) vs 4.5 (1)	0.12
		1 vs 2	3 (1) vs 4.5 (1)	0.05
Resource Developer	Senior Residents	0 vs 1	4.5 (1) vs 3.5 (1.25)	0.02
		0 vs 2	4.5 (1) vs 4 (1)	0.03
		1 vs 2	3.5 (1.25) vs 4 (1)	0.01
	Assistant Professors	0 vs 1	4 (1) vs 3 (1)	0.06
		0 vs 2	4 (1) vs 4 (1)	0.09
		1 vs 2	3 (1) vs 4 (1)	0.04
	Senior Professors	0 vs 1	4.5 (1) vs 4 (1)	0.09
		0 vs 2	4.5 (1) vs 4.5 (1)	0.11
		1 vs 2	4 (1) vs 4.5 (1)	0.07

For the role of Planner, senior residents and assistant professors revealed significant differences, between categories 0 and 1, and 1 and 2. This indicates a profound disparity between the perceived importance of the role and their current and future commitments. Among Assistant Professors, we observed significant difference among categories (0, 1 and 2) in the roles of role model and facilitator. This suggests that Assistant Professors may foresee a heightened commitment to being role models and facilitators.

No statistically significant variations were observed for Senior Professors across any roles, with all p-values staying above the 0.05 threshold. This suggests a relatively consistent perception and commitment to their roles, possibly reflecting a stabilization in their career stage. The role of examiner results was uniform across all categories and teacher types. This may point to a generally accepted understanding of the role's importance and required commitment among medical teachers.

For Senior Residents and assistant professors, statistically significant differences were observed between all category pairs. In the case of Senior Professors, the median scores were relatively high across all categories, with Categories 0 and 2 having a slightly higher median of 4.7 (IQR=1-5) compared to Category 1, which had a median of 4.2 (IQR=0.5-5). However, none of these differences were statistically significant. This suggests that while there are some discernible patterns in how different categories perform across roles, these patterns are not uniform across the teacher types. Notably, Senior Residents showed significant variations across categories, whereas Senior Professors were relatively consistent (Table 5).

**Table 5:** Comparison of overall score between categories and teachers' subcategories. Mann-Whitney U Test

Overall, Role Categories	Senior Residents	Assistant Professors	Senior Professors
Category 0 Median (IQR)	4.5 (1-5)	4.5 (1-5)	4.7 (1-5)
Category 1 Median (IQR)	4.0 (0.5-5)	4.0 (0.5-5)	4.2 (0.5-5)
Category 2 Median (IQR)	4.5 (1-5)	4.5 (1-5)	4.7 (1-5)
P-value (0 vs 1)	0.01	0.05	0.10
P-value (0 vs 2)	0.02	0.07	0.12
P-value (1 vs 2)	0.01	0.04	0.11

## Discussion

Medical education has been witnessing a slow change over the past few years with emphasis shifting from teacher-directed learning to students’ self-directed study [8]. This changing paradigm of learning has led to an increase in the complexity of roles played by the teacher in medical education. Harden and Crosby in 2000 gave 12 roles of a teacher divided into six areas of activities such as information provider, role model, facilitator, assessor, planner and resource developer [1].

Information provider

Teachers’ roles as information provider in the form of lecturer in a classroom or clinical teacher in the hospital or community settings constitute the traditional roles that are the most widely used instructional methods [9]. In a study conducted in three medical and dental colleges in Islamabad, the majority of medical teachers perceived their most important role as information providers in both lecture and clinical settings. This perception aligns with traditional curricula that allocate maximum time for long didactic lectures, where students act as passive listeners [6]. In contrast, a study involving faculty members of Mashhad University of Medical Sciences categorized the role of information provider into two sub-roles: lecturer in the classroom and teacher in the clinical setting. Faculty members were asked to score the importance of each role on a 1-10 scale. Although the study did not explicitly state the scores for the role of information provider, it laid out a framework for evaluating the importance of this role among others like role model, facilitator, and examiner [8]. When comparing results across different institutes and even with Dundee University, the predominant role was still that of an information provider. However, the study also noted that senior faculty members gave more importance to roles with educational expertise, whereas less experienced teachers were more focused on traditional roles with medical expertise [6]. These studies collectively suggest that while the role of the information provider is universally acknowledged as crucial, its relative importance can vary depending on the educational environment, level of teaching experience, and even cultural factors.

Role model

Role modeling in the context of on-the-job role model during clinical rounds or as a teacher in the classroom has a much more lasting impact on the students compared to other methods of teaching [10]. Certain characteristics like enthusiasm for teaching, reasoning skills, effective communication with active student involvement and a holistic view of the patient have been found to be desirable by students in their teacher as a good role model [11]. This is perceived differently across various studies. In a study from Islamabad, the role of a teacher as a role model was rated as equally important as that of a mentor, with 75% and 64% importance, respectively. The study distinguishes between the two roles, stating that mentors are older persons in an organization who actively guide younger people, while role models teach primarily by example. Role models may have brief contact with students but often play many roles simultaneously [6]. In another study, the average score for "on-the-job role model" was 9.47, the highest among all roles. This indicates the faculty's awareness of the significant influence role models can have on students' future career choices [8].

Facilitator

Due to the shift of the concept of learning to being student-centred, the role of a teacher as a learning facilitator in problem-based learning and as a mentor or guide or trusted counsellor is gaining importance [12,13]. In one study, the role of a learning facilitator received a mean score of 4.1 on a scale of 1 to 10. This score was comparable to other roles like mentor or personal advisor, which also received a mean score of 4.1. Another study assigned a mean score of 8.53 to the roles of facilitation of learning and mentoring/tutoring. The study noted that the lower score, in comparison to role modeling, could be attributed to either a lack of time or an imprecise definition of mentoring in the faculties' minds. It was also mentioned that "learning facilitator" is an important role in "problem-based learning," but faculty might not have assigned a very high score to this role because they have not yet involved themselves in this method of teaching [6, 8].

Examiner

Assessment also has moved ahead from the traditional examination system. Teachers need to assess both the students (summative as well as formative) and the course curriculum. Assessment of the course curriculum can be performed through student feedback, peer evaluation or assessment of the product of an educational course [14,15]. In one study, the role of planning or participating in student exams was included in the questionnaire but did not receive a specific mean score. However, senior faculty members, such as professors and associate professors, were noted to give more importance to roles with educational expertise, including assessment and curriculum evaluation. In another study participants assigned the highest score to "planning or participating in student exams". This suggests that they view assessment as the most important responsibility the university wants them to undertake. The study also noted that "Curriculum evaluation" is an important responsibility, and faculty were aware of its significance, assigning a similar score to this role as for "planning or participating in student exams" [6, 8].

Planner

The two categories of teachers’ roles that have received the least enthusiasm are the functions of planner and resource developer. The role of planner includes both curriculum planning and meticulous description of the courses that comprise the curriculum [16,17].

In a study, the role of a "Curriculum Planner" received a mean score of 4.1 on a scale of 1 to 10. This score was similar to other roles like mentor, personal advisor, and learning facilitator, which also received a mean score of 4.1. The study suggests that while faculty members acknowledge the importance of planning, it is not considered the most critical role in their educational setting [6].

In another study, a low score given to these roles indicates that faculty may be unaware of the significant impact that planning activities have on learning. The study also suggests that lack of time might be a contributing factor. In a study in Iran, only 50% of faculty members had prepared written lesson plans for the courses they taught. The faculty stated that high workload, low impetus, and lack of knowledge about writing lesson plans were among the reasons preventing them from planning effectively [8].

Resource developer

Teachers require to spend a significant amount of time in the creation of resource material both in traditional workbooks and computer-based learning [18]. In addition to the resource material, study guides need to be prepared that guide the students on the expected learning outcomes, the resources available and opportunities for self-assessment, especially for topics that are already covered in books or other resources [19]. In one of the studies, this role received a mean score of 3.9, which is relatively low compared to other roles like information provider and role model. It suggests that the role of a resource developer is acknowledged but not highly prioritized. This could be due to a lack of awareness about the importance of educational resources other than textbooks and lectures, or it could reflect the educational culture and priorities of the institutions involved [6].

Inter-category comparison for teacher subcategories

The present study revealed that there is a significant difference in perceived future commitment among senior residents and assistant professors for certain roles. This not only highlights the dynamic nature of role perception but also indicates that younger faculty members are optimistic about their future role engagement. This also suggests that younger faculty may not be static in their role commitments; they anticipate evolving in their various roles, underscoring the importance of continual faculty development efforts. Contrastingly, Senior Professors did not show any statistically significant variations in their roles, possibly pointing to a stabilization in their career stage and a fixed approach towards their teaching roles. It might be beneficial to explore if this stabilization is due to a lack of stimuli for change or a conscious choice based on years of experience.

Hence, the roles of a teacher, as described by Harden and Crosby, are all-encompassing and adequate importance needs to be given to all these roles for efficient student learning. In the last two decades, few studies have evaluated the teachers’ perception of these 12 roles and the acceptability of the newer roles that have been defined by Harden and Crosby.

Once made aware of the roles of a teacher, the prioritization of these roles changed from traditional to modern roles, thus highlighting the impact of awareness of these new roles [4]. After an extensive search of the available literature, we could find only a single Indian study evaluating the dental teachers’ perception of the 12 roles of a teacher. The study reported that teachers in dental education perceived their role as information provider in clinical setting, on-the-job role model and classroom teaching as most important, both in their present and future commitment [20]. However, a similar analysis has not yet been carried out among medical teachers in India.

The present study is, to the best of our knowledge, the first such evaluation of awareness and commitment towards the framework of teachers’ role described by Harden among medical teachers in India. The median scores of perception of various roles as being important to medical teaching showed only a little variation. This is slightly at variance to the earlier Indian study among dental teachers who described greatest importance to the role as clinical teacher followed by on-the-job role model [20]. A consistent observation in the earlier studies as well as the present study is the lowest score given to roles like making study guide, curriculum planner and course organizer [6,8,20]. These low scores may be attributed to multiple factors: unawareness of the faculty regarding the impact of a well-designed curriculum on students learning; lack of time or expertise in writing a lesson plan and low impetus for such roles [21]. Though the roles of a teacher, as described by Harden and Crosby, are widely accepted internationally, some of the roles do not seem to receive as much attention among the faculty members. In our setting, the same may be ascribed to the undue emphasis placed on theoretical knowledge and examination grades rather than on practical skills. This in turn leads the medical teachers to focus on classroom teaching and other such roles than on proper curriculum planning and being a learning facilitator.

The degree of present and preferred future commitment to each of these roles has not been assessed in many studies. The study by Deshpande among dental teachers in India showed that the personal present commitment was less when compared to the perceived importance of various roles in dental teaching [20]. In the present study, a statistically significant difference was observed between the perception of importance to medical teaching, personal current commitment and preferred future commitment, except for the role as lecturer in the classroom setting. Inter-category comparison also showed significant differences between the perceived importance and present commitment (p<0.05) as well as between present and future commitment (p<0.05). This comparison assumes importance for planning of suitable medical faculty development programs (FDP) by colleges and universities through medical education departments [22]. A recent report from Saudi Arabia highlighted the need for FDP workshops and seminars with emphasis on the development of learning objectives and tailoring the course specification to match the same. The study also emphasized the requirement of a feedback system from the students as well as a peer-review system with inbuilt mentoring [23]. A recent review reiterated the need of faculty development in Indian medical education as per the changing role of teachers for effective student learning [24].

The main strength of the present study is the fact that this was the first evaluation of teachers’ roles among medical faculty in India. Since we used the categorization described by Harden and Crosby, the present and future personal commitments of teachers to each of these roles were also known. This information would be useful in designing the FDPs in our setting. However, a few limitations do need mention. Firstly, the number of participant teachers in the present study was low, mainly because of the recent incorporation of medical undergraduate teaching at our hospital. This led to limitations of data analysis among categories of gender, professions and academic experience. Being a single institutional study, the results may not be generalizable to other medical schools in India. However, a comparison with the literature shows that our findings were not too different from the other studies. Hence, these results can be suitably utilized by other medical institutions as a starting point for their FDPs.

## Conclusions

In conclusion, the present study is the first-of-its-kind analysis from India of medical teachers’ perception of varied roles described in AMEE Guide No. 20. The results of our study highlight the lack of perceived importance of certain vital roles of a teacher among our medical school faculty. Though this is a mono-institutional study, we would like to strongly recommend that universities as well as medical colleges across the country should undertake teachers’ training workshops in order to encourage their faculty for improvement in roles that are perceived as less important. It may be beneficial for educational institutions to adopt a longitudinal approach in their faculty development initiatives, along with regular self and peer-assessments, at different stages of career to re-evaluate and align their role commitments accordingly. Such efforts shall pave the way for significant progress in the quality of medical education in the country.
